# Comparative Analysis of Genomics and Proteomics in the New Isolated *Bacillus thuringiensis* X022 Revealed the Metabolic Regulation Mechanism of Carbon Flux Following Cu^2+^ Treatment

**DOI:** 10.3389/fmicb.2016.00792

**Published:** 2016-05-27

**Authors:** Meifang Quan, Junyan Xie, Xuemei Liu, Yang Li, Jie Rang, Tong Zhang, Fengjuan Zhou, Liqiu Xia, Shengbiao Hu, Yunjun Sun, Xuezhi Ding

**Affiliations:** ^1^Hunan Provincial Key Laboratory of Microbial Molecular Biology-State Key Laboratory Breeding Base of Microbial Molecular Biology, College of Life Science, Hunan Normal UniversityChangsha, China; ^2^Laboratory of Medicine Engineering, College of Medicine, Hunan Normal UniversityChangsha, China

**Keywords:** *Bacillus thuringiensis*, genome sequencing, annotation, proteomics, comparative analysis, poly-β-hydroxybutyrate

## Abstract

*Bacillus thuringiensis* (*Bt*) X022 is a novel strain isolated from soil in China, and showed strong insecticidal activity against several *Lepidopteran* pests. In this work, we performed whole genome sequencing of this *Bt* strain using the next-generation sequencing technology, and further conducted a comparative analysis with the proteomics data of the specific spore-release period based on LC-MS/MS approach. The *Bt* X022 genome consisted of one circular chromosomal DNA and seven plasmids, which were further functionally annotated using the RAST server. Comparative analysis of insecticidal substances showed that X022 contained genes coding for three Cry proteins (Cry1Ac, Cry1Ia and Cry2Ab) and a vegetative insecticidal protein (Vip3A). However, three insecticidal crystal proteins (ICPs) (Cry1Ca, Cry1Ac and Cry1Da) were detected by proteomics in the spore-release period. Moreover, a putative biosynthetic gene cluster and the metabolic pathway for poly-β-hydroxybutyrate in *Bt* X022 were deduced based on the comparative analysis of genomic and proteomic data, which revealed the metabolic regulation mechanism of carbon flux correlated with increased production of ICPs caused by Cu^2+.^ Hence, these results provided a deeper understanding of the genetic background and protein expression profile of *Bt* X022. This study established a foundation for directed genetic modification and further application of this new isolated *Bt* strain.

## Introduction

*Bacillus thuringiensis* (*Bt*) is a known Gram-positive entomopathogenic bacterium, and products based on it are currently the most widely used biopesticides (Sanahuja et al., 2011). The insecticidal activities of this bacterium are mainly due to the crystal proteins produced during sporulation, which typically consist of various ICPs, including Cry and Cyt protoxins (Ibrahim et al., 2010). Importantly, the ICPs of *Bt* exerted insecticidal activities toward an extensive array of insects, such as *Lepidoptera*, *Diptera*, and *Coleoptera* (van Frankenhuyzen, 2009, 2013). With its advantages of high efficiency, target specificity and environmental compatibility, *Bt* has attracted much attention of researchers all over the world.

However, the high-efficiency synthesis of ICPs and subsequent parasporal crystals formation process is not clear yet. These processes appear to be precisely regulated by a functional protein network with spatio-temporal and orderly occurring characteristics which remains to be elucidated (Baum and Malvar, 1995). On the other hand, as the result of increasing emergence of resistance to the existed toxins of the *Bt* strains, it is still necessary to explore novel insecticidal gene resources of *Bt* to control agricultural pests effectively (Coates and Siegfried, 2015; Guo et al., 2015).

More recently, combination of genomics and proteomics emerged as a valuable approach for solving these problems. Since the first complete whole genome sequencing of *Bt* strain serovar konkukian 97-27 in 2004 (Han et al., 2006), the number of sequenced genomes has increased sharply over the past decade, among which, 15 *Bt* strains have been completely sequenced up to now. The whole 6.3 Mb genome of *Bt* 4.0718 has been sequenced by our laboratory (Rang et al., 2015). It comprises a circular chromosome of 5.6 Mb encoding 6747 open reading frames (ORFs) and seven circular plasmids. On the other hand, recent development of MS-based proteomics has enabled the detection of total proteins from different growth stages of a microorganism, which can therefore add new accurate interpretation to the genomic sequences, such as new hypothetical proteins identification and the regulatory pathways of some cellular processes (Wang et al., 2013a,b; Zhang et al., 2014). Such a combination of genomics and proteomics stratege has been successfully implemented in a wide variety of organisms for validation and correction of predicted genomic coding information (Gupta et al., 2008; Borchert et al., 2010; Yang et al., 2014). Therefore, this combination approach may be a useful tool to address the problem of mining new insecticidal substances and identification of functional proteins interacting network that associated with spore and parasporal crystal formation in *Bt*.

*Bt* X022 is a novel strain isolated from the soil in China by our laboratory. This strain produces diamond parasporal crystals and exerts strong insecticidal activities against *Spodoptera exigua* and *Helicoverpa armigera*. Referring to the morphology under microscope and 16S rRNA gene sequenced, we preliminarily determined that *Bt* X022 belonged to *Bt* subsp. *kurstaki*. A study by Liu et al. (2015) found that the ICP production of *Bt* X022 was efficiently increased when a appropriate concentration of Cu^2+^ was added. The mechanisms involved were further explored by performing a comparative proteomic analysis of the total proteins for X022 in spore-release period based on LC-MS/MS approach.

In this study, we firstly performed whole genome sequencing of *Bt* X022 using next-generation sequencing (NGS) technology. Secondly, the resulting genomic sequence data were assembled and functional annotated using the Rapid Annotation using Subsystem Technology (RAST) server. Moreover, an in-depth comparative analysis of the genomic sequence data and the proteomic results from Liu et al. (2015) for strain X022 was further performed; with the object of mining new insecticidal gene and functional protein resources, and further revealing the complicated regulating mechanisms correlated with increased ICP production and insecticidal toxicity of *Bt* X022.

## Materials and Methods

### Bacterial Strains and Cultural Conditions

The novel isolated strain *Bt* X022 (CCTCC No. M2014158) was previously described by Liu et al. (2015). *Bt* X022 cells was cultured overnight at 30°C with agitation at 200 rpm in liquid LB medium, comprised of polypeptone 10 g; yeast extract 5 g and NaCl 10 g/L (pH 7.6). Subsequently, 400 μL of the overnight culture was transfered into a 300 mL shaker-flask containing 20 mL fresh LB medium and grown for approximately another 3 h at 30°C until the optical density at 600 nm (OD_600_) reached 1.0.

### Genomic DNA Preparation

Total DNA of *Bt* X022 was extracted with Bacterial DNA Kit (Omega Bio-Tek Inc., Lilburn, GA, USA) according to the manufacturer’s instructions. Briefly, 3 mL bacterial culture at an OD_600_ of 1.0 was harvested and the cell pellets were resuspended in 180 μL TE buffer containing 5 mg/mL lysozyme and incubated at 30°C for 10 min. Thereafter, the pellet digested cells were collected and subjected to effect complete lysis through incubation at 55°C in a shaking water bath with 200 μL Buffer BTL containing 25 μL Proteinase K solution. Five microliter RNase A was then added and incubated at room temperature for 30 min, followed by treatment with 220 μL Buffer BDL and incubation at 65°C for 10 min. Two hundred and twenty microliter absolute ethanol was then added and mixed thoroughly, and the entire sample was transfered to a DNA binding column and centrifuged at 10,000 × *g* for 1 min to bind DNA. After washed twice with washing buffer, the total DNA was finally eluted from the column and collected into a new 2 mL tube. Subsequently, the extracted total DNA sample was quantitated using a Nanodrop 2000 (Thermo scientific, Waltham, MA, USA) and subjected to electrophoresis examination in 0.7% agarose gel for 30 min at 120 V and another 10 h at 20 V. Finally, The samples packed on dry ice were sent to HuaDa Genomic Co. Ltd (Shenzhen, Guangdong, China) for genome sequencing.

### Genome Sequencing and Assembly

The whole genomic sequencing of *Bt* X022 was completed with Illumina Hiseq 2000 by HuaDa Genomic Co. Ltd (Shenzhen, Guangdong, China). Briefly, total DNA sample of X022 was firstly randomly broken into smaller pieces of no longer than 800 bp by sonication with Bioruptor instruments. Next, the generated fragmented DNA are blunted using a mixture of T4 DNA Polymerase, Klenow DNA Polymerase and T4 PNK, followed by further ligation of specific adapter sequences to the ends. The resulted fragments were purified, amplified by PCR, and were finally used for library construction and sequencing. For genome assembly, the closest species *Bt* serovar *kurstaki* str. HD73 was used as the reference genome, and the resulting 244 scaffolds were firstly compared with the genome sequence of HD73 on line using the blast tool^1^ respectively to determine the relative position of each scaffold in the chromosome. Thereafter, these scaffolds were assembled in order using Gene Construction Kit 3.0, and the gaps between contigs were filled by PCR amplification.

### Genome Annotation and Analysis

Automatic genome annotation was performed using the RAST server^2^. Genome structural analysis contains tandem repeat analysis, non-coding RNA analysis (including rRNA, tRNA, and sRNA), and gene prediction. Genome functional analysis comprises conventional gene annotation, gene family analysis and phylogeny analysis. And the predicted ORFs were further functionly annotated by employing GO, KEGG, Swiss-Prot, NR and COG.

### Proteomic Analysis Based on LC-MS/MS Approach

The extraction of total bacterial proteins from X022 cells, trypsin digestion and 2D-LC-MS/MS analysis, database search and functional classification processes were previously described by Liu et al. (2015).

### PCR Detection of Toxin Genes in *Bt* X022

The presence of putative genes for proteins Cry1Ca and Cry1Da were confirmed by PCR. The primers used for *cry1Ca* and *cry1Da* amplification were designed based on the N-terminal conservative region of the published sequences in *Bt* strains^3^. Meanwhile, three genes encoding for insecticidal proteins (Cry1Ia, Cry2Ab and Vip3A) were also detected. All primers used for detection of these five genes were listed in **Table 1**. The PCR amplification was performed in 20 μL volume containing 2 μL 10 × buffer, 200 ng genomic DNA, 1.6 μL dNTP mixture (2.5 mM), 50 pmol primers and 1.25 U Ex Taq DNA polymerase. The obtained PCR products were subjected to an electrophoresis examination in 1.0% agarose gel for 30 min at 120 V, and finally sent to Thermo Fisher Scientific (China) Co. Ltd (Shanghai, China) for sequence confirmation.

**Table 1:** Primers for PCR detection of toxin genes.

Gene	Primer name	Primer Sequence(5′→3′)	Product size (bp)
l*cry1Da*	cry1Da-F	CTGTAGCAGACATTTCATTAGG	
l	cry1Da-R	TGTCAAGGCCTGTAATATATGAA	1020
l*cry1Ca*	cry1Ca -F	AATCAAAATCAATGCATACCTTAC	
l	cry1Ca -R	TAAAGTCCTAAATACCGGTCCAT	1080
l*vip3Aa*	vip3Aa -F	ATGGCATTTATGGATTTGCCACT	
l	vip3Aa -R	CCACAATCATCTTTGCATCTTCAT	987
l*cry1Ia*	cry1Ia -F	ATGAAACTAAAGAATCAAGATAAGC	
l	cry1Ia -R	ATACTTCTCTTGTAAGTTGGGCT	886
l*cry2Ab*	cry2Ab -F	ATGAATAGTGTATTGAATAGCGGA	
l	cry2Ab -R	TAATTTGAATTAACTTGGAAAAG	917

### RNA Extraction and Quantitative Real Time RT-PCR

Total RNA was extracted from the bacterial cells harvested at the time point of 24 h cultivation using TRIzol Reagent (Invitrogen Biotechnology Co., Ltd., Shanghai, China). Following digestion of the residual genomic DNA by DNase I (Thermo Fisher Scientific Co. Ltd., Shanghai, China), 1 μg of total RNA was reverse transcribed to cDNA utilizing RevertAid^TM^ First Strand cDNA Synthesis Kit (Thermo Fisher Scientific Co. Ltd., Shanghai, China) according to the manufacturer’s instruction. The two-step real-time RT-PCR analysis were conducted using Power SYBR^^®^^ Green PCR Master Mix (Thermo Fisher Scientific Co. Ltd., Shanghai, China) in a CFX Connect Real-Time PCR Detection System (Bio-Rad Laboratories, Inc., Hercules, CA, USA), as previously described (Liu et al., 2015). qRT-PCR primers used in this study were summarized in **Table 2**. 16S rRNA was used as a normalization control for gene expression.

**Table 2:** Primers for real-time RT-PCR analysis.

Gene	Primer name	Primer Sequence (5′→3′)	Product size (bp)
16SrRNA	16S-F	CTTGACATCCTCTGAAAACCCTA	107
	16S-R	GACTTAACCCAACATCTCACGAC	
P-type ATPase 1	ATPase1 -F	GTTAGATGGTGTGAACAAAGCGAC	108
	ATPase1 -R	CTTCGTAATCGCACTCTTCATTTC	
P-type ATPase 2	ATPase2-F	ATTGATGAAGCAGCAATCACG	133
	ATPase2-R	GGAATAATGTTTGATCGCTTGG	
Multicopper oxidase	Oxidase -F	CAGCTACAACACCATTGAAGGT	126
	Oxidase -R	TTCTGGACCAGGAGATGAGC	
Copper resistance protein D	Resistance -F	GGCTACGTCCCACAAATCGA	120
	Resistance -R	CTTGATCGAACCGAGGTTGC	
cutC	CutC -F	GCAGCTGGTGTAGTATTAGGCG	111
	CutC -R	ATCTATCGCACGGTGGTACGT	
cry1Ia	cry1Ia -F	ATGGCCTAAGGGGAAAAATCA	156
	cry1Ia -R	ACCCAACTTTCAAGCGAATCAT	
cry2Ab	cry2Ab -F	ATTGACAGGGCTGCAAGCA	156
	cry2Ab -R	GTATCCTTGCATCTGGAACTGG	

*P-type ATPase 1, was located at the position of 3700335-3697915 bp in the genome of Bt X022. P-type ATPase 2, was located at the position of 532169-530244 bp in the genome of Bt X022*.

## Results

### Genome Assembly and Functional Annotation of *Bt* X022

Whole genome sequencing of *Bt* X022 was completed with the Illumina Hiseq 2000 sequencing platform, which resulted in a total of 706 Mb clean data containing 8,151,360 reads. And all the paired reads (~96.23% of the total) were further assembled into 244 scaffolds (470 contigs in total) using Short Oligonucleotide Alignment Program (version 2.04). The above scaffolds were assembled in order based on the reference genome of closest species *Bt* serovar *kurstaki* str. HD73 in NCBI. The gaps between scaffolds were filled by PCR amplification. Assembly results revealed that *Bt* X022 contained a circular chromosome DNA, which consisted of 170 contigs with a total size of 5,460,989 bp and an average G + C content of 32.67%. The longest contig contained 580,476 bp and the length of N50 contig was 32,069 bp. The draft genome for strain X022 also revealed that eight plasmids with high homology to pDNA-involved, pCT72, pHT8-2, pBTHD789-4, pK1S1, pHT73, pCT14 and pCT281 (failed assembly) were possibly harbored (**Table 3**).

**Table 3:** Summary of the plasmids well assembled.

Number	Size	CDSs	Closest homologous plasmids
1	14881	28	pBTHD789-4
2	12866	24	pCT14
3	69351	89	pCT72
4	10716	14	pDNA-involved
5	8509	9	pHT8-2
6	77278	72	pHT73
7	5598	6	pK1S1

**FIGURE 1:**
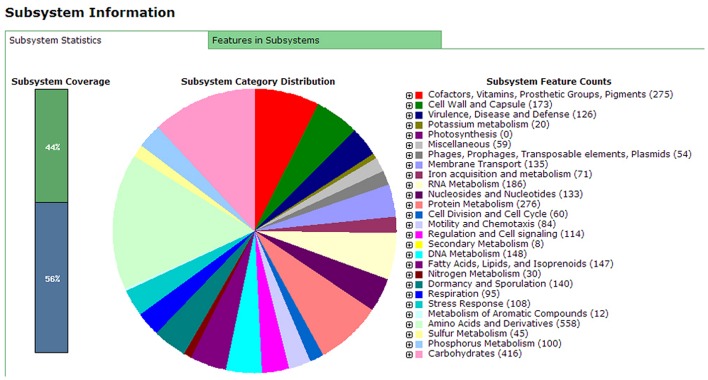
**Subsystems category distribution of *Bacillus. thuringiensis* subsp. serovar *kurstaki* strain X022 revealed by genome annotations based on the RAST server.** The pie graph indicates the subsystem distribution statistics of Bt X022 strain. Each color represents a subsystem category with the feature counts of which listed on the right of the graph.

**FIGURE 2:**
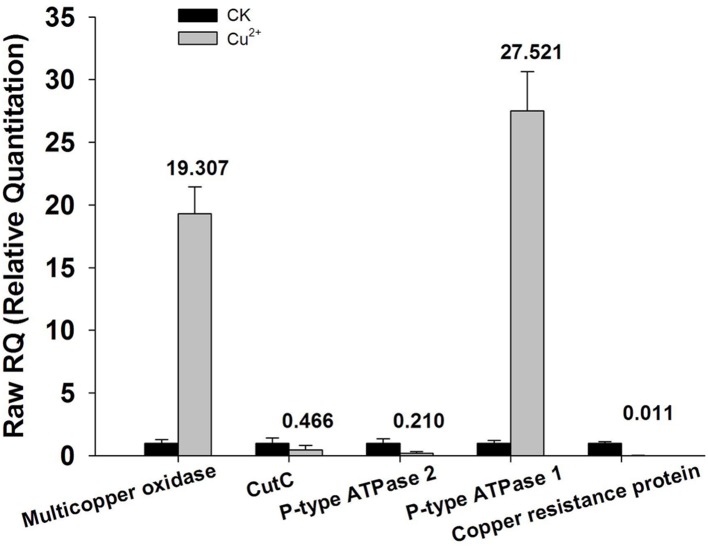
**qRT-PCR analysis of encoding genes responsible for copper homeostasis.** Total RNA extracted from bacterial cells harvested at the time point of 24 h cultivation was reverse-transcribed into cDNA. Relative mRNA expression levels of the five selected genes (P-type ATPase 1and 2, multicopper oxidase, Copper resistance protein D and cutC) were analyzed by qRT-PCR using primers specific for each of the genes. 16S rRNA was used as an internal control. Data are calculated from four independent determinations of mRNA abundance in each sample.

Annotation results of the chromosome DNA sequence based on RAST server showed that the total 170 contigs of the X022 strain contained 5646 coding sequences (CDS) and 148 ncRNAs. The length of total CDS was 4,538,678 bp, which corresponded to 83% of the whole chromosome DNA sequence. The subsystem statistics of the genome based on annotation by RAST server were presented in **Figure 1**: Genes associated with amino acids and derivatives (558 ORFs), carbohydrates (416 ORFs), and protein metabolism (276 ORFs) were ranked the first three abundant categories among the whole subsystem (**Figure 1**). Genome annotation results showed that *Bt* X022 elaborated copper homeostatic systems which comprises Copper-translocating P-type ATPase, Multicopper oxidase, Copper resistance protein D and Cytoplasmic copper homeostasis protein CutC. These functional proteins attempted to maintain the normal physiological activities of bacterial cells under certain levels of extracellular copper. To analyze the gene expression differences between the two conditions (fermentation medium and Cu^2+^-containing condition), five genes coding for copper homeostasis(P-type ATPase1, P-type ATPase2, Multicopper oxidase, Copper resistance protein D and CutC) were subjected to real-time RT-PCR analysis. We found that both mRNA levels of P-type ATPase1 and Multicopper oxidase were upregulated, and the other three genes were downregulated in the Cu^2+^-containing condition (**Figure 2**).

**FIGURE 3:**
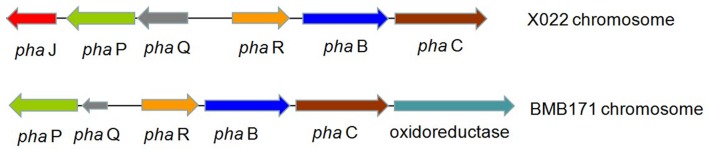
**Putative biosynthetic gene cluster for PHB in *Bt* X022 and its comparison with that in *Bt* BMB171 (Chen et al., 2012).** The cluster in *Bt* X022 consisted of six PHB-related genes within a 3755 bp region: *phaJ* (encoding 3-hydroxybutyryl-CoA dehydratase), *phaP* (encoding a phasin protein), *phaQ* (encoding a PHB-responsive repressor controlling expression of phaP and phaQ), *phaR* (encoding a subunit of PHB synthase), *phaB* (encoding acetoacetyl-CoA reductase), and *phaC* (encoding polyhydroxyalkanoic acid synthase).

Encoding genes responsible for PHB biosynthesis and metabolism, which were associated with the increased ICP production caused by Cu^2+^, were also found located in the chromosome. The biosynthetic gene cluster for PHB in *Bt* X022 consisted of six ORFs, namely, *phaJ*, *phaP*, *phaQ* (a repressor of *phaP*), *phaR* (a subunit of PHB synthase), *pha B* (acetoacetyl-CoA reductase), and *pha C* (PHB synthase). The genetic organization of this biosynthetic gene cluster for PHB in *Bt* X022 was slightly different from that in *B. megaterium* and *Bt* strain BMB171 (Chen et al., 2012). However, in *Bt* strain BMB171, this biosynthetic gene cluster was composed of *phaP*, *phaQ*, *phaR*, *pha B*, *pha C* and an oxidoreductase (**Figure 3**).

Preliminary analysis also presented that the well assembled seven plasmids carried 242 predicted CDSs, with a total length of 152,490 bp for CDS regions (**Table 1**). Hence, the genomic features of *Bt* X022 and its comparative analysis with that of *Bt* 4.0718 were listed in **Table 4**. The chromosomal size of *Bt* 4.0718 was about 5.6 Mb, which contained 6797 predicted CDSs. These results indicated that *Bt* 4.0718 exhibited a more complicated genetic background compared to *Bt* X022.

**Table 4:** An comparative analysis of the Bt X022 and Bt 4.0718 genome features.

Features	*Bt* X022	*Bt* 4.0718
Chromosome size	5,460,989 bp	5,641,982 bp
Number of plasmids	7	6
Predicted CDSs	5888	6797
Hypothetical genes	1592	1826
ncRNAs	148	94
Predicted insecticidal genes	4	6

### Preliminary Comparative Analysis of Genomics and Proteomics Data for *Bt* X022

**FIGURE 4:**
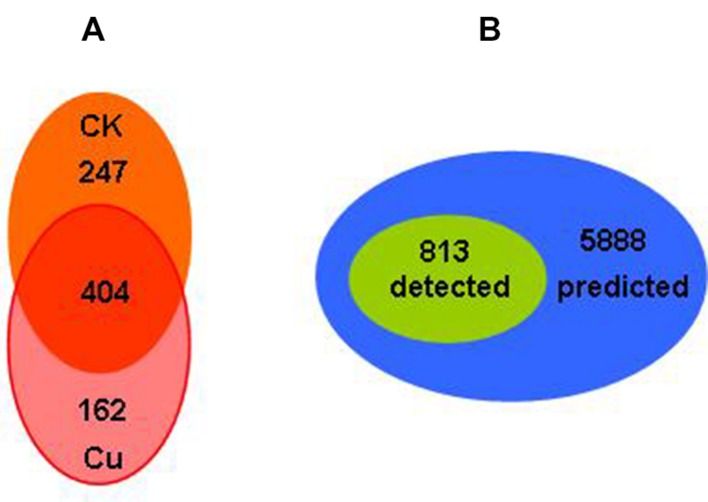
**Comparative analysis of genomics and proteomics data for Bt X022. (A)** Venn diagram indicating the total proteins detected in the two culture conditions in the spore-release period of X022 (Liu et al., 2015). **(B)** Venn diagram illustrating the comparison of the total proteins detected in the spore-release period of X022 (813 proteins) and the predicted proteomics of X022 (5888 CDSs).

The total protein expressions profile in *Bt* X022 by proteomic analysis based on 2D-LC-MS/MS approach were described previously by Liu et al. (2015). A total of 813 proteins in X022 were identified in the spore-release period (44 h): 651 and 566 proteins were identified in fermentation medium (CK) and Cu^2+^-containing condition, respectively. Among these proteins, 404 proteins were commonly detected in both conditions (**Figure 4A**). According to the functional classification system reported by Huang et al. (2012), these identified proteins of *Bt* X022 were classified into seven functional groups by using the Gene Ontology (GO), KEGG and BioCyc metabolic pathway database of Uniprot KB: Small molecular metabolism (39.32%), Macromolecular metabolism (24.42%), Unknown function (18.13%), Cell processes (7.83%), Regulation (2.76%), Environmental information processing (6.76%) and Extra-chromosomal proteins (0.77%). We preliminarily estimated that the total 813 proteins detected in both conditions, which constitute the expressed genome in spore-release period for *Bt* X022, accounted for 13.8% of the total predicted proteome encoded by the whole genome (**Figure 4B**).

### Comparative Analysis of Insecticidal Substances

**FIGURE 5:**
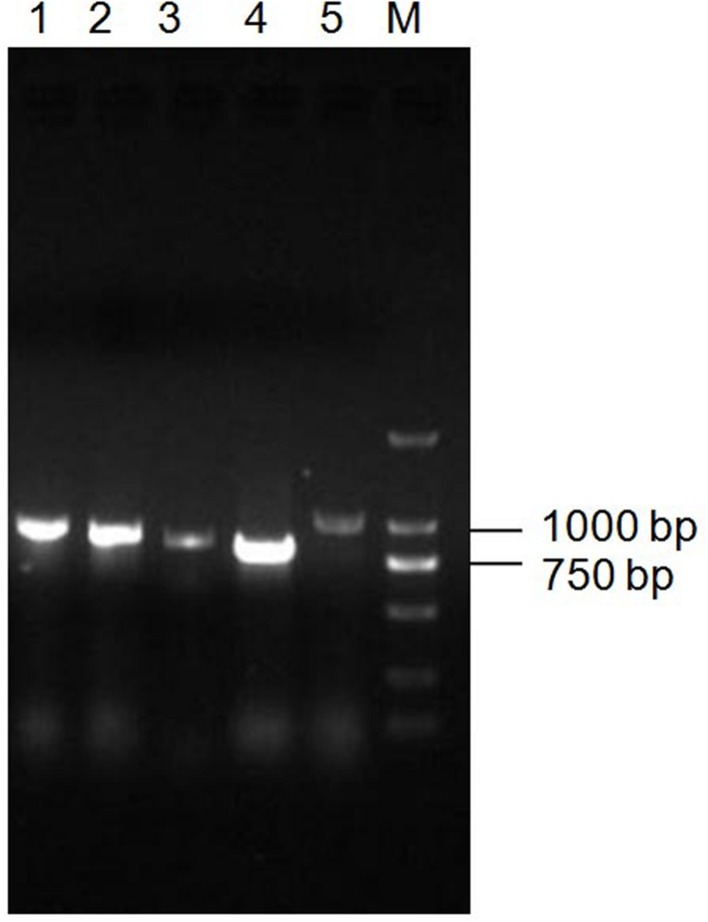
**PCR detection of toxin genes in *Bt* X022.** The genes encoding for insecticidal proteins (Cry1Ca, Cry1Da, Cry1Ia, Cry2Ab and Vip3A) were detected by PCR using primers specific for each of the genes. The obtained PCR products were subjected to an examination by agarose gel electrophoresis. Line 1, *cry1Da*; Line 2, *vip3A*; Line 3, *cry2Ab*; Line 4, *cry1Ia*; Line 5, *cry1Ca*; M, DL2000 DNA marker.

Since *Bt* X022 was found to exert a strong insecticidal activity against several target insects, it was tempted to mine the functional gene resources that were related with its entomopathogenic characteristics based on the above genomic and proteomic data. The assembled genome for strain X022 contained one complete ICP gene which showed a 100% pairwise amino acid identity with pesticidal crystal protein cry 1Ac from *B. cereus* (**Table 5**). Moreover, we found that this strain also included two other complete crystal protein gene (*cry1Ia* and *cry2Ab*) and a vegetative insecticidal protein gene (*vip3A*) in two scaffolds of the failed assembled plasmid pCT281 by screening the comprising scaffolds with BLASTx analysis. The putative *cry1Ia* gene encoded a shorter insecticidal protein of 719 amino acids (aa) with 100% pairwise identity to the 746 aa protein of other *Bt*. Insecticidal genes *cry2Ab* and *vip3A* exhibited 100 and 99% pairwise amino acids identity to those of *B. cereus* and *Bt*, respectively. The annotated genome for strain X022 also included up to 20 CDSs which were all located in the chromosome and related with insecticidal activity, including non-hemolytic enterotoxin A, immune inhibitor A, chitinase, hemolysin BL, hemolysin III, cytotoxin K and SpoIISA-like protein. However, the 2D-LC-MS/MS results showed that three ICPs: Cry1Ca, Cry1Ac and Cry1Da were detected and revealed to be mainly expressed during the spore-release period of strain X022. To confirm the presence of putative genes for crystal proteins (Cry1Ca and Cry1Da), an PCR detection of these five genes (*cry1Ca*, *cry1Da*, *cry1Ia*, *cry2Ab* and *vip3A*)was conducted. All these PCR amplifications generated products with the expected size (**Figure 5**). And sequencing of *cry1Ca* and *cry1Da* PCR products further confirmed their presence in *Bt* X022. We also try to confirm the expression of *cry1Ia* and *cry2Ab* by qRT-PCR. However, transcription of *cry1Ia* and *cry2Ab* in these two culture conditions were not detected (data not shown).

**Table 5:** Comparative description of the insecticidal substances detected in genomic annotation and/or proteomic data for strain X022.

Closest homolog from genomic annotation	Location	LC-MS/MS detection
Cry 1Ac	Plasmid pHT73	+
Cry1Ia, Cry2Ab and Vip3A	Plasmid pCT281	- / Cry1Ca and Cry1Da
Immune inhibitor A	Chromosome	+
Chitinase	Chromosome	+
Non-hemolytic enterotoxin A	Chromosome	–
Non-hemolytic enterotoxin lytic component L1	Chromosome	–
hemolysin III	Chromosome	–
Hemolysin BL lytic component L1	Chromosome	–
Hemolysin BL lytic component L2	Chromosome	–
Hemolysin BL binding component precursor	Chromosome	–
Cytotoxin K	Chromosome	–
SpoIISA like protein	Chromosome	–

*+/-, the insecticidal activity substance can be/cannot be detected*.

### Comparative Analysis of PHB Synthesis and Metabolism Regulation

Liu et al. (2015) have reported that Cu^2+^ addition caused significant improvement of crystal protein production were probably associated with carbon flow changes, which was indicated by the downregulation of glycan biosynthesis and metabolism-related proteins. Genome annotation combined with KEGG database search results revealed that up to 20 CDSs that coded for proteins participated in PHB synthesis and metabolism located in the chromosome (**Table 6**). However, only PhaJ (3-hydroxybutyryl-CoA dehydratase), Hbd (3-hydroxybutyryl-CoA dehydrogenase), EchA (Enoyl-CoA hydratase), AtoB (Acetyl-CoA acetyltransferase), HADH (3-hydroxyacyl-CoA dehydrogenase), PhaP and PhaR were detected by LC-MS/MS in the spore-release period of this strain.

**Table 6:** Comparative analysis of the proteins participated in PHB metabolism and regulation in genomics and proteomics for stain X022.

Function description	Genome annotation results	LC-MS/MS results
poly(3-hydroxybutyrate) depolymerase	3-Oxoadipate enol-lactonase(phaZ)	–
3-hydroxybutyrate dehydrogenase	D-β-hydroxybutyrate dehydrogenase(BDH)	+
Acetoacetyl-CoA synthetase	Acetoacetyl-CoA synthetase(acsA)	–
Acetoacetyl-CoA reductase	phbB	–
Polyhydroxyalkanoic acid synthase	phaC	–
3-hydroxybutyryl-CoA dehydratase	phaJ	+
3-hydroxybutyryl-CoA dehydrogenase	hbd	+
Enoyl-CoA hydratase	Enoyl-CoA hydratase(echA)	+
Acetyl-CoA acetyltransferase	Acetyl-CoA acetyltransferase(atoB)	+
3-hydroxyacyl-CoA dehydrogenase	3-hydroxyacyl-CoA dehydrogenase(HADH)	+
D-β-hydroxybutyrate permease	Polyhydroxybutyrate metabolism	–
phaP protein	phaP	+
a PHB-responsive repressor controlling expression of phaP and phaQ	PhaQ	–
Polyhydroxyalkanoate synthesis repressor	PhaR	+
Polyhydroxybutyrate metabolism	Short chain fatty acids transporter	–

*+/-, the PHB metabolism-related proteins can be /cannot be detected*.

**FIGURE 6:**
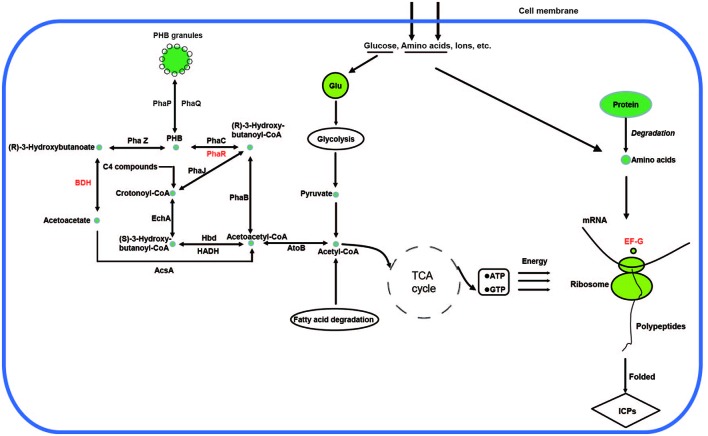
**Poly-β-hydroxybutyrate biosynthesis and metabolism regulation of X022.** The related proteins in the biosynthetic and metabolic pathways were obtained from genomic annotation data of X022 and further integrated into a network according to the KEGG database (http://www.genome.jp/kegg-bin/show_pathway?ko00650$+$K00023). The protein names in red represent the significantly upregulated proteins in response to Cu ^2+^ treatment as reported by Liu et al. (2015).

Accordingly, a network for PHB biosynthesis and metabolism regulation of X022 was finally drawn based on the related enzymes annotated from genome and KEGG database (**Figure 6**). **Figure 6** indicated that in the logarithmic phase, the excess carbon supply fluxed to the PHB biosynthetic pathway through an important intermediate metabolite acetyl-CoA. However, when X022 cells grew into the stationary phase and the carbon and energy source around the extracellular environment were gradually exhausted, the reserved carbon resources could be reutilized through degradation of PHB and convertion into acetyl-CoA, finally entering into the tricarboxylic acid (TCA) cycle to release and provide energy for the important physiological processes, such as sporulation and ICP synthesis. Importantly, Liu et al. (2015) found that two PHB metabolism-related proteins, namely, PhaR and 3-hydroxybutyrate dehydrogenase (BDH), were upregulated in response to Cu^2+^ treatement, accompanied by an increased ICP production. It was therefore speculated that Cu^2+^ might have changed the direction of carbon flow in strain X022, finally resulting in more energy reserved and further reuse for ICP synthesis.

## Discussion

In this study, we performed whole genome sequencing of the new isolated *Bt* X022, and further conducted a comparative analysis with the proteomics data of the specific spore-release period, with the aims of comprehensively understanding the genetic information and its total proteins expression profile in new strain X022. Firstly, whole genome sequencing of X022 was completed using the Illumina Hiseq 2000 sequencing platform. The X022 genome was finally assembled into one circular chromosome DNA and seven complete plasmids by homology searching in the blast server using the closest species *Bt* serovar *kurstaki* str. HD73 as the reference genome and gap filling with PCR amplification.

More recently, NGS technologies (Shendure and Ji, 2008; Metzker, 2010) provide a unique way to obtain whole genomic sequences of microorganisms rapidly and economically, which has led to the remarkable increase in the number of microbial genome sequences. However, in addition to the simple sequence acquisition, an in-depth explanation to the microbial genome depends more on a further proper annotation of the sequence data, which include the function and genomic localization of all CDSs. RAST is a fully-automated service for high-quality annotation of complete or nearly complete bacterial genomes (Aziz et al., 2008; Overbeek et al., 2014; Brettin et al., 2015). In this work, X022 complete genome was automatically annotated using the RAST server, and results showed that the chromosome DNA of X022 encoded 5646 CDS in total. Among these CDSs predicted, a series of functional genes, such as those associated with copper homeostasis and PHB biosynthetic cluster were found. And the following qRT-PCR analysis of five genes coding for copper homeostasis (P-type ATPase1, P-type ATPase2, Multicopper oxidase, Copper resistance protein D and CutC) showed that both mRNA levels of P-type ATPase1 and Multicopper oxidase were up-regulated, while the other three genes were down-regulated in the Cu^2+^-containting condition. The results of P-type ATPase1 and Multicopper oxidase were in consistent with the research reported previously (Rensing et al., 2000; Stoyanov et al., 2001). P-type ATPase was responsible for pumping copper from the cytoplasm into the periplasm (Rensing et al., 2000). Multicopper oxidase could convert periplasmic Cu(I) to Cu(II) (Grass and Rensing, 2001). Upregulation of these two proteins was possibly able to reduce the toxic effects of copper on the bacterial cells.

Besides these successfully annotated genes, a large proportion of CDSs encoding short proteins exhibiting no or low identity with homologs of other species (hypothetical protein) were also found. These proteins made up 25.6% of the total CDSs. Similarly, the high proportion of hypothetical protein was found in the seven plasmids annotated. This large amounts of genes without any known homologs existing in the genome was also reported in other species (Dieterich et al., 2008; Borchert et al., 2010). However, the function and expression of these genes remain to be explored and demonstrated by other approaches in the future.

Moreover, a comparative analysis between the genomics and proteomics data of X022 was conducted. The 2D-LC-MS/MS results showed that a total of 813 proteins were detected in spore-release period of X022 in the two conditions (CK and Cu^2+^ supplemented), which represented 13.8% of the predicted proteomics encoded by the whole genome of X022 (5646 for chromosome and 242 for plasmids). Compared to the reference strain HD73 which contained only one endotoxin gene *cry1Ac* in plasmid pHT73 (Liu et al., 2013), the genome of X022 was shown to harbor 3 cry-endotoxin genes (cry1Ac, cry1Ia and cry2Ab) and one vegetative insecticidal protein gene vip3A. Among these four genes found, *cry1Ac* was in a plasmid with high homology to pHT73, whereas *cry1Ia*, *cry2Ab* and *vip3A* were found in two scaffolds of the failed assembled plasmid pCT281. Interestingly, the proteomics results identified that strain X022 mainly expressed three ICPs, namely, Cry1Ac, Cry1Ca and Cry1Da. However, encoding genes for the 2D-LC-MS/MS detected Cry1Ca and Cry1Da proteins were not found in the genomics data yet. We speculated that these two genes might be located in the missing part of some certain failed assembled plasmids. And the following PCR detection experiment using primers specific for each of the genes confirmed our speculation (**Figure 5**). On the other hand, the Cry1Ia protein was a secreted delta-endotoxin produced by several *Bt* strains during vegetative growth and could not form a classic crystal like other Cry proteins (Dammak et al., 2011; Varani et al., 2013). Thus, it was not surprising that Cry1Ia and Vip3A proteins were not detected during the spore-release period of X022. The absence of Cry2Ab in the total protein sample of X022 was probably due to other reasons. A qRT-PCR analysis was conducted to confirm the expression of *cry1Ia* and *cry2Ab* in mRNA level. Unfortunately, transcription of these two genes were not detected at the time point of 24 h cultivation in normal or Cu^2+^-containing condition (data not shown). Moreover, in addition to these insecticidal proteins, the X022 genome also contained no less than 20 encoding genes associated with insecticidal activities. However, only Immune inhibitor A and Chitinase were successfully detected by 2D-LC-MS/MS during the spore-release period.

In this study, we also screened the annotated genomic data of X022 for the encoding genes associated with PHB metabolism. As expected, in addition to the biosynthetic gene cluster for PHB, another 10 genes related to PHB metabolism were found at different sites of the chromosome. Compared with the genome annotation results, the 2D-LC-MS/MS results showed that eight proteins (PhaJ, BDH, Hbd, EchA, AtoB, HADH, PhaP and PhaR) were detected in the total protein sample of strain X022 in the spore-release period. Although Liu et al. (2015) had present related data to speculate that carbon flow changes, which was indicated by upregulation of two PHB metabolism-related proteins PhaR and BDH, may contribute to the increased ICP production caused by Cu^2+^ addition in X022 culture. And several studies had demonstrated a positive correlation between PHB production and delta-endotoxin synthesis (Navarro et al., 2006). The mechanism by which copper influenced the carbon flow changes in Bt cells has not been revealed.

According to the metabolic pathways of PHB deduced, under appropriate conditions, PHB synthesis was initiated with conversion of two acetyl CoA molecules to aceto-acetyl CoA by Acetyl-CoA acetyltransferase (atoB). Macomber and Imlay (2009) had reported that certain concentrations of Cu^+^ and Cu^2+^ could both directly inactivate fumarase A *in vivo* and *in vitro*. As we know, the TCA cycle is a key metabolic pathway, which starts with the oxidation of acetyl-CoA derived from carbohydrates, fats and proteins degradation (Wolfe and Jahoor, 1990). As Fumarase is one of the important enzymes involved in TCA cycle, it was speculated that the suppression of Fumarase activity by Cu^2+^ might led to inhibition of TCA cycle and more acetyl CoA originated from glycolysis, fatty acid degradation or other pathways fluxing to PHB synthesis. Carbon source was eventually assimilated and stored in cells in the form of PHB granules. In addition, when the sporulation and ICPs synthesis were proceeding and massive energy was needed, the PHB reutilization was initiated by depolymerization of PHB by 3-Oxoadipate enol-lactonase (*phaZ*), a novel intracellular PHB depolymerase identified in *Bt* (Tseng et al., 2006). The carbon resource released from PHB degradation finally flowed into TCA cycle through the intermediate compound acetyl CoA. Upregulation of BDH and PhaR protein resulted in the increased deposit and reutilization of PHB, and consequently led to the increased ICP production, as indicated by upregulation of another protein translation elongation factor G (EF-G) (Liu et al., 2015). These results revealed the metabolic regulation mechanism of carbon flux correlated with increased ICP production caused by Cu^2+^.

Collectively, a series of insecticidal substances and functional proteins associated with PHB metabolism and their encoding genes were found in X022 by the combined use of genomics and proteomics approaches. These results provided a deeper understanding of the genetic background and protein expressions profile in strain X022, which would greatly facilitate further directed genetic modification and other applied research of this new isolated Bt strain.

## Author Contributions

XD, LX, SH, and YS designed the experiments. MQ, JX, XL, YL, and JR performed the genomic DNA extraction and genomic data analysis. MQ, JX, XL, YL, JR, TZ, and FZ carried out the proteomic analysis. MQ and XD draft the manuscript. LX, SH, and YS revised the manuscript. All authors read and approved the final manuscript.

## Conflict of Interest Statement

The authors declare that the research was conducted in the absence of any commercial or financial relationships that could be construed as a potential conflict of interest.

## Footnotes

1 http://blast.ncbi.nlm.nih.gov/Blast.cgi

2 http://rast.nmpdr.org/

3 http://www.lifesci.sussex.ac.uk/home/Neil_Crickmore/Bt/

## Abbreviations

2D-LC-MS/MS: two-dimensional liquid chromatography-tandem mass spectrometry
BDH: 3-hydroxybutyrate dehydrogenase
EF-G: elongation factor G
ICP: insecticidal crystal proteins
PHB: poly-β-hydroxybutyrate
